# Abstract of Proceedings of the Buffalo Medical Association

**Published:** 1866-10

**Authors:** T. M. Johnson

**Affiliations:** Sec’y.


ART. II—*Abstract of Proceedings of the Buffalo Medical Association.*

TUESDAY EVENING, Sept. 4th, 1866.

The meeting was called to order by the President.

Members present: Drs. Gould, Rochester, Jansen, Boardman, Wetmore, Gleason, Camerling, Miner, Little, Trowbridge, Lothrop and Johnson.

The report of the proceedings of the July meeting was read and adopted.

Dr. BOARDMAN reported an operation in which he used Richardson's mode of local anæsthesia. On the 2d of June by means of a spray apparatus, obtained from Mr. Peabody, he applied ether spray for about thirty-five seconds to a small tumor situated in the skin on the left deltoid. The skin was partly blanched. He removed the tumor by an elliptical incision one and a half inches in length, and closed the wound with two sutures and straps. There was some bleeding from the cut, but not as much as usual. The patient, a nervous female, was engaged in conversation with the head turned away to avoid the fumes of the ether. As he was introducing the last suture she asked why he did not commence the operation. On being told to look, that the operation was about completed, she would hardly believe the evidence of her own eyes, declaring that she had felt nothing. Two-thirds of the wound closed by first intention, but she taking a severe cold on the fourth day, the wound inflamed and most of the cicatrix gave way. The patient now states that she has had more or less severe neuralgic pain in the wound ever since. Dr. B. thought the wound would have healed kindly save for the cold. He is not prepared to state whether or not the neuralgic pain was caused by the local anæsthesia. He was much pleased with the effect of the spray, and thinks that in superficial operations it may become very useful, but although more serious operations might be performed without pain, by the repeated application of the spray to the cut, yet he would prefer the use of the general anæsthesia produced by inhalation in the latter cases. He does not think that Richardson ever intended to have the parts frozen, for if this was done, not only would the operation be much more difficult, but the wound would not heal as kindly.

Dr. Rochester reported a case of severe urethral hæmorrhage. A man was riding upon a bare-backed horse, and was violently jolted. He soon perceived blood flowing very freely from the urethra. A practitioner was called in who pronounced it hæmorrhage from injury of the kidney. Ice was applied to the loins and abdomen, and the patient was enjoined not to urinate. Dr. Rochester was called at midnight—found a distended bladder which was blocked up by a clot and had to be evacuated with a catheter. Very profuse hæmorrhage ensued, the blood being arterial in color, and discharged per saltum. The hæmorrhage was readily controlled by pressure upon the perineum. The persulphate of iron in doses of ten drops, was ordered every four hours, and the patient directed to urinate whenever he felt inclined to do so. In three days the man was discharged. The case was mentioned as interesting and instructive in itself, and as affording a curions instance of careless diagnosis, and consequent bad treatment in the outset.

Dr. Miner said he had been invited to call the attention of the Association to specimens of the following pharmaceutical preparations prepared by Howell and Onderdonk, of New York: Elixir calisaya, iron and bismuth; elixir pyrophosphate iron; elixir pyrophosphate iron and soda; elixir valerianate ammonia; elixir valerianate ammonia and quinine; elixir valerianate strychnia, citrate and tannate bismuth, and solution protoxide of iron. He considers them all elegant and agreeable preparations, especially the preparation of bark and iron, and bark, iron and bismuth. Bark alone, as thus prepared, is one of those medicines that amuse sick people until they get well, and is not otherwise very efficacious in the cure of disease. The preparations of iron and bark are good and useful. The simple elixir calisaya, as formerly prepared and quite largely prescribed, is a very fanciful preparation, and of insignificant medicinal value.

Dr. Gleason observed that the formulæ for the preparations spoken of by Dr. Miner was published by Caswell, Mack & Co., and that he believes that the druggists of Buffalo can put up the various preparations of iron calisaya and bismuth, as well as the druggists of New York and Philadelphia, and at much less expense to consumers.

Dr. Boardman said that in this connection he would like to call the attention of the Association to a pliable machine-spread belladona plaster, kept ready for use by prescription druggists. He had used it considerably of late as an application to painful swellings, buboes, boils, stitch or pain in the side, scrofulous joints, etc. Would not use it on an abraided surface. Had never observed the constitutional effects from local application and thinks it an excellent application and local anodyne in the above and similar diseases.

Dr. Miner said that twenty years ago it was quite fashionable to employ belladona externally to relieve pains and inflammations, especially was it common for the purpose of drying up or suppressing the secretion of milk, and preventing mammary abscess; but when it is remembered how these plasters are made, the extract mixed largly with resin, so that but little, if any of the medicine comes in contact with the skin, it is doubtful if great effect is produced; it will not dry up the milk and is not a good narcotic or anodyne. It will dilate the pupil even when used in small quantity, and is very useful for that one purpose, and of little or no value for anything else. The preparation spoken of by Dr. Boardman is elegant and the best in use.

Dr. Boardman is fully satisfied that it did allay pain in the cases that he mentioned before.

Dr. Rochester was glad that Dr. Miner had introduced the preparations of bark, iron, etc. They are elegant and agreeable, but do not think them quite as reliable or as good as the ferruginous preparations we were accustomed to use formerly, and do not believe it proper to prescribe such preparations put up in New York and Philadelphia, and scattered over the country in the style of patent medicines. Doctors ought to sustain good druggists at home and not prescribe these fancy and more expensive articles.

Dr. Gould thought that the druggists here charge quite high enough for medicines, and had heard it intimated that they allowed in some cases a percentage to physicians. Had never supposed however, that to be the case.

Dr. Gleason said that there was no association among druggists here, and no uniformity in prices.

Dr. Miner said that no respectable druggist would offer a percentage to physicians, and believed that the druggists in Buffalo were generally conducting their business in this respect honorably and honestly. He would regard allowing percentage to physicians as dishonest for both druggist and physician, and had not known either a respectable druggist to offer it, or physician to accept it.

Dr. Rochester said, it is quite common in New York for physicians to get a percentage on their perscriptions. He had never known of but one case here; that was a man who was accustomed to this course in his native country, he being a foreigner and not now in business.

Dr. Rochester moved that a committee of three be appointed to examine the Constitution and By-Laws and ascertain the expense of printing and binding the same in proper form, said committee to report at the next regular meeting. Carried.

The President appointed as such committee Drs. Boardman, Trowbridge and Wetmore.

Report on prevailing diseases being in order, Dr. Boardman reported an unusual amount of diarrhea, and said that although the disease is usually very easily checked it is unusually persistent.

Dr. Rochester had seen more diarrhea and cholera-morbus than ever before and a few cases of dysentery—no cholera.

Adjourned. T. M. Johnson, Sec'y.

